# High risk of early sub-therapeutic penicillin concentrations after intramuscular benzathine penicillin G injections in Ethiopian children and adults with rheumatic heart disease

**DOI:** 10.1371/journal.pntd.0009399

**Published:** 2021-06-11

**Authors:** Ezra B. Ketema, Nigus Z. Gishen, Abraha Hailu, Abadi Leul, Abera Hadgu, Kiflom Hagos, Samual Berhane, Temesgen Tsega, Madhu Page-Sharp, Timothy ME Davis, Brioni Moore, Kevin T. Batty, Jonathan Carapetis, Sam Salman, Laurens Manning

**Affiliations:** 1 Department of Medical Biochemistry and Molecular Biology, School of Medicine, College of Health Sciences, Mekelle University, Mekelle, Ethiopia; 2 Department of Medicine, DebreBerhan University, DebreBerhan, Ethiopia; 3 Department of Internal Medicine, School of Medicine, College of Health Sciences, Mekelle University, Mekelle, Ethiopia; 4 Department of Pediatrics and Child Health, School of Medicine, College of Health Sciences, Mekelle University, Mekelle, Ethiopia; 5 Department of Pharmacology, School of Pharmacy, College of Health Sciences, Mekelle University, Mekelle, Ethiopia; 6 Department of Medical Microbiology, School of Medicine, College of Health Sciences, Mekelle University, Mekelle, Ethiopia; 7 School of Pharmacy and Biomedical Sciences, Curtin University, Bentley, Western Australia, Australia; 8 Faculty of Health and Medical Sciences, University of Western Australia, Perth, Western Australia, Australia; 9 Wesfarmers Centre for Vaccines and Infectious Diseases, Telethon Kids Institute, Perth, Western Australia, Australia; 10 Department of Infectious Diseases, Perth Children’s Hospital, Perth, Western Australia, Australia; 11 Clinical Pharmacology and Toxicology, PathWest, Western Australia, Australia; 12 Department of Infectious Diseases, Fiona Stanley Hospital, Murdoch, Australia; National University of Singapore, SINGAPORE

## Abstract

**Introduction:**

Intramuscular benzathine penicillin G (BPG) injections are a cornerstone of secondary prophylaxis to prevent acute rheumatic fever (ARF) and rheumatic heart disease (RHD). Uncertainties regarding inter-ethnic and preparation variability, and target exposure profiles of BPG injection are key knowledge gaps for RHD control.

**Methods:**

To evaluate BPG pharmacokinetics (PK) in patients receiving 4-weekly doses in Ethiopia, we conducted a prospective cohort study of ARF/RHD patients attending cardiology outpatient clinics. Serum samples were collected weekly for one month after injection and assayed with a liquid chromatography-mass spectroscopy assay. Concentration-time datasets for BPG were analyzed by nonlinear mixed effects modelling using NONMEM.

**Results:**

A total of 190 penicillin concentration samples from 74 patients were included in the final PK model. The median age, weight, BMI was 21 years, 47 kg and 18 kg/m^2^, respectively. When compared with estimates derived from Indigenous Australian patients, the estimate for median (95% confidence interval) volume of distribution (V/F) was lower (54.8 [43.9–66.3] l.70kg^-1^) whilst the absorption half-life (t_1/2-abs2_) was longer (12.0 [8.75–17.7] days). The median (IQR) percentage of time where the concentrations remained above 20 ng/mL and 10 ng/mL within the 28-day treatment cycle was 42.5% (27.5–60) and 73% (58.5–99), respectively.

**Conclusions:**

The majority of Ethiopian patients receiving BPG as secondary prophylaxis to prevent RHD do not attain target concentrations for more than two weeks during each 4-weekly injection cycle, highlighting the limitations of current BPG strategies. Between-population variation, together with PK differences between different preparations may be important considerations for ARF/RHD control programs.

## Author summary

Rheumatic heart disease (RHD) is the most common form of acquired heart disease in Ethiopia across all age groups. Monthly benzathine penicillin G (BPG) injections remains the cornerstone of secondary prophylaxis to prevent recurrent acute rheumatic fever (ARF) and progression to RHD, but data supporting the optimal dose and timing of BPG injections is a major knowledge gap for National RHD control programs, particularly amongst the highest risk groups. In this study we measured penicillin concentrations in Ethiopian patients receiving regular BPG injections. It demonstrates that that most Ethiopian patients receiving BPG as secondary prophylaxis to prevent RHD do not maintain target concentrations for more than two weeks during each 4-weekly injection cycle. This study highlights the limitation of the current BPG dosing recommendations and underpin global calls for improved penicillin formulations and better dosing and delivery strategies.

## Introduction

Rheumatic heart disease (RHD) affects more than 33.4 million people worldwide, especially children and young adults from low and middle income countries and vulnerable populations living in high income countries, and results in 319,000 premature deaths annually. [1] The administration of benzathine penicillin G (BPG) injections remains the cornerstone of secondary prophylaxis to prevent recurrent acute rheumatic fever (ARF) and RHD. [2–4] BPG is superior to oral penicillin and non-penicillin antibiotics in preventing skin or throat infections caused by *Streptococcus pyogenes* (Group A Streptococcus; Strep A) which precede episodes of ARF. [5,6] Based on efficacy and pharmacokinetic (PK) data from studies conducted in the 1950s, most guidelines still recommend a 900 mg dose (1.2 million international units [MU]) of BPG every 3–4 weeks administered by deep intramuscular (IM) injection for a minimum of 10 years. [3,4,7] After injection, the benzylpenicillin G (penicillin) dissociates from the benzathine moiety and is absorbed from the injection site into the blood stream.

It is widely assumed that protective efficacy of BPG is determined by the time that penicillin concentrations measured in plasma or serum remain >20 ng/mL (0.02 mg/L). [8]. However, this has never been tested in formal PK-pharmacodynamic (PK-PD) studies in humans and there are limited data relating to BPG PK in populations at highest risk of ARF. [9] Current dosing regimens are based on data from studies of healthy male military recruits or children conducted >50 years ago. [10–14] Extrapolating more recent population PK models, also performed in military recruits, [15] to patients with ARF/RHD in low income settings may be inappropriate due to differences in age, body composition, drug formulations and disease effects.

Our recent population PK study of BPG in Indigenous Australian children and young adults with RHD [16] and a randomized cross-over study comparing IM with subcutaneous (SC) administration of BPG have provided new insights into BPG pharmacology, particularly in relation to the effects of body composition and SC administration. [17] These models highlight the importance of a slow absorption profile of penicillin from the depot site as a key determinant for subsequent plasma concentrations and the apparent slow clearance from the central compartment. Delayed absorption in obese or overweight patients, presumably due to inadvertent SC administration, highlights the potential for wide variability in BPG PK in patients with different body composition. Furthermore, differences in BPG crystal sizes within and between different formulations may also influence absorption kinetics. [18]

BPG is a critical medication in Ethiopia where RHD is the most common form of acquired heart disease across all age groups, accounting for more than half of cardiovascular disease and a quarter of cardiovascular deaths among children and young adults. [19,20] A recent community-based echocardiographic survey identified 37.5 (95% confidence interval [CI_95_] 26.9–51.8) cases of RHD per 1000 children/young adults, a rate >1000-fold higher than published data from resource rich countries. [21]

Since there are no contemporary PK data for BPG in the Ethiopian population, determining the optimal doses and timing of BPG injection remain the key challenges for National RHD control programs. This is of particular concern as Strep A and ARF relapses have been documented in children adherent to regular BPG, in both the Ethiopian context and in Indigenous Australians. [22,23] How body composition, formulation and dose impacted on these results is unclear because details relating to body weight, dose, and formulation were not provided and plasma penicillin concentrations were not measured.

Due to the significant concerns regarding the adequacy of contemporary BPG prophylaxis strategies in Ethiopia and the lack of locally-derived PK data to inform programmatic changes, we aimed to fill these knowledge gaps by undertaking a PK study in a population of patients receiving regular BPG for RHD in northern Ethiopia. The findings may have implications for anthropometrically and epidemiologically similar populations.

## Methods

### Ethics statement

Ethical clearance was obtained from the Ethics Review Committee of the College of Health Sciences (1053/2017), Mekelle University, Ethiopia. Written informed consent was obtained from adult patients and each child’s parent or guardian. Assent was also obtained from participants aged >12 years. Adult patients and parents/ guardians consented to serum samples being sent abroad for further analysis. Exemptions were obtained from the University of Western Australia (UWA) and Curtin University for receipt and analysis of the samples (Ref#: RA/4/20/4915). A material transfer agreement for the transfer of human serum samples was signed between Curtin University and Mekelle University representatives as per the Ministry of Science and Technology, Ethiopia guidelines. Subsequently, sample export permission was obtained from the Food, Medicine and Health Care Administration and Control Authority (FMHACA) of Ethiopia. A permit to import human fluids and tissues were also granted to Curtin University (Permit #: 0001267471) by the Department of Agriculture and Water Resources, Australian Government.

### Study setting and design

This prospective cohort study was conducted at Ayder Comprehensive Specialized Hospital from February to October 2018. The hospital is one of the largest government referral hospitals located in Mekelle, Tigray Region, Northern Ethiopia. It provides both referral and basic patient care services to 9 million people in its catchment areas of the Tigray, Afar and North-eastern parts of the Amhara Regional States. We undertook a population PK study of a cohort of ARF/RHD patients followed during a single 4-week interval between routinely scheduled BPG injections for measurement of serum penicillin concentrations.

### Patient recruitment

Patients with ARF/RHD attending adult and pediatric cardiac outpatient clinics were eligible for recruitment. A total of 40 children and 66 adults with confirmed ARF or RHD and receiving BPG injections for ≥3 months prior to the initiation of the study were enrolled. Only patients willing to adhere to study procedures, including an additional 2–3 visits for blood collections before their next injection, were included. Patients who did not have regular follow up at the study site, who did not volunteer to return for blood collection visits, or who had <3 months of prophylaxis were excluded from the study.

### Socio-demographic, clinical and laboratory data collection

Information on sociodemographic variables (including age, sex and family size), medication use (duration, compliance, frequency and type of secondary prophylaxis) and clinical data (history of recurrence, co-morbidities and other RHD associated complications) was obtained by trained nurses in collaboration with attending physicians from eligible participants using structured questionnaires and review of medical records. Diagnosis of AFR/RHD recurrences were confirmed by the treating physicians. We defined recurrent ARF symptoms based on the modified Jones’ criteria. [24]

### Administration of BPG

According to the local guidelines, IM BPG injection should be administered every 28 days for secondary prophylaxis against ARF recurrences. Patients weighing >27 kg receive 900mg (1.2MU) while those <27 kg receive 450 mg (0.6 MU) on each occasion. A powdered formulation from an Ethiopian generic manufacturer (Ethiopian Pharmaceuticals Manufacturing Factory, Addis Ababa, Ethiopia) was administered following reconstitution according to the manufacturer’s instructions. Experienced nurses, trained in the administration of BPG delivered each IM injection and inspected the vials after the dose had been drawn up to ensure that the full dose had been given.

### Blood sampling

Patients were requested to return on Day 14 and 21 following the BPG injection for blood collection. This sampling schedule was changed during the study to include an additional Day 7 visit when possible. Travel and food expenses for patients for return visits were covered by the research team. The last blood collection was at the Day 28 appointment before the next monthly BPG injection (also the day 0 time point at steady state). Approximately 5 mL of venous blood was collected into BD vacutainer tube (Becton Dickinson, USA), separated by centrifugation and stored at -70°C before transportation on dry ice to Australia for penicillin assay. Creatinine measurements were not available. Blood samples were drawn by experienced registered nurses and sent promptly to central laboratory by porters for serum preparation and storage by senior laboratory technologists.

### Measurement of penicillin concentrations

Penicillin concentrations were assayed using a validated liquid chromatography-mass spectroscopy (LC-MS) assay. [25] Quality control samples for accuracy and inter- and intra-day variation for across concentrations between 2.5–1000 ng/mL confirmed the assay performance. The assay limits of quantification (LOQ) and detection (LOD) were 1 and 0.5 ng/mL, respectively.

### Data analysis and pharmacokinetic modelling

Data were analysed using R [26] and presented as medians (inter-quartile range [IQR]) or proportions. Log_e_ plasma concentration-time datasets for BPG were analysed by nonlinear mixed effects modelling using NONMEM (v 7.2.0, ICON Development Solutions, Ellicott City, MD, US) with an Intel Visual FORTRAN 10.0 compiler. First order conditional estimation with interaction (FOCE with INTER) was used.

An abbreviated model building process was undertaken. Given the sparse concentration vs time dataset available, the model structure from a previous population PK model in patients receiving BPG for secondary prophylaxis was used. [16] This model incorporated allometric scaling (to standard weight of 70kg) and consists of a single compartment for BPG with first order elimination (fixed according to literature values) and two sequential phases of absorption for the BPG salt. Because no concentration data until 7 days after the dose were available, the faster absorption half-life (t_½, abs-1_) was fixed to the value determined in the prior model. A sensitivity analysis was performed for the final model to test this assumption and the effect on estimated model parameters. Inter-individual variability (IIV) as well as correlations between IIV terms were included where supported by the data. The influence of body mass index (BMI) using a reported cut-off of 25 kg/m^2^ [16] and each unit between 20–30 kg/m^2^, on absorption profile was re-evaluated with the present data. Finally, a sensitivity analysis was also performed to assess the impact of fixing the elimination constant from the central compartment (k_el_) to 1.32 h^-1^.70kg^-1^ as in previous studies. [16]

For model evaluation, plots of observed vs individual- and population-predicted values, and time *versus* population weighted residuals (WRES), were first assessed. A bootstrap using Perl speaks NONMEM (PSN) with 1,000 samples was performed, and the parameters derived from this analysis summarised as median and 2.5th and 97.5th percentiles (CI_95_) to facilitate evaluation of model parameter estimates. In addition, visual predictive checks (VPCs) were performed with 1,000 datasets simulated from the final models. The observed 10th, 50th, and 90th were plotted with their respective simulated 90% CIs to assess the predictive performance of the model and to evaluate any major bias. Shrinkage of population variability parameters and residual variability was calculated. [27]

## Results

A total of 106 patients, 66 from adult cardiac outpatient clinic and 40 from the pediatric cardiac unit, were enrolled. Due to missing data, 32 participants were excluded from the PK analysis, including 27 without a documented BMI and two for whom gender was not recorded. Gender and BMI are required to estimate individual fat free mass, a key component of the PK model. Two patients had higher concentrations recorded at Day 21 than at Day 14, which were implausible and their data were also excluded from the analysis.

**Fig 1:**
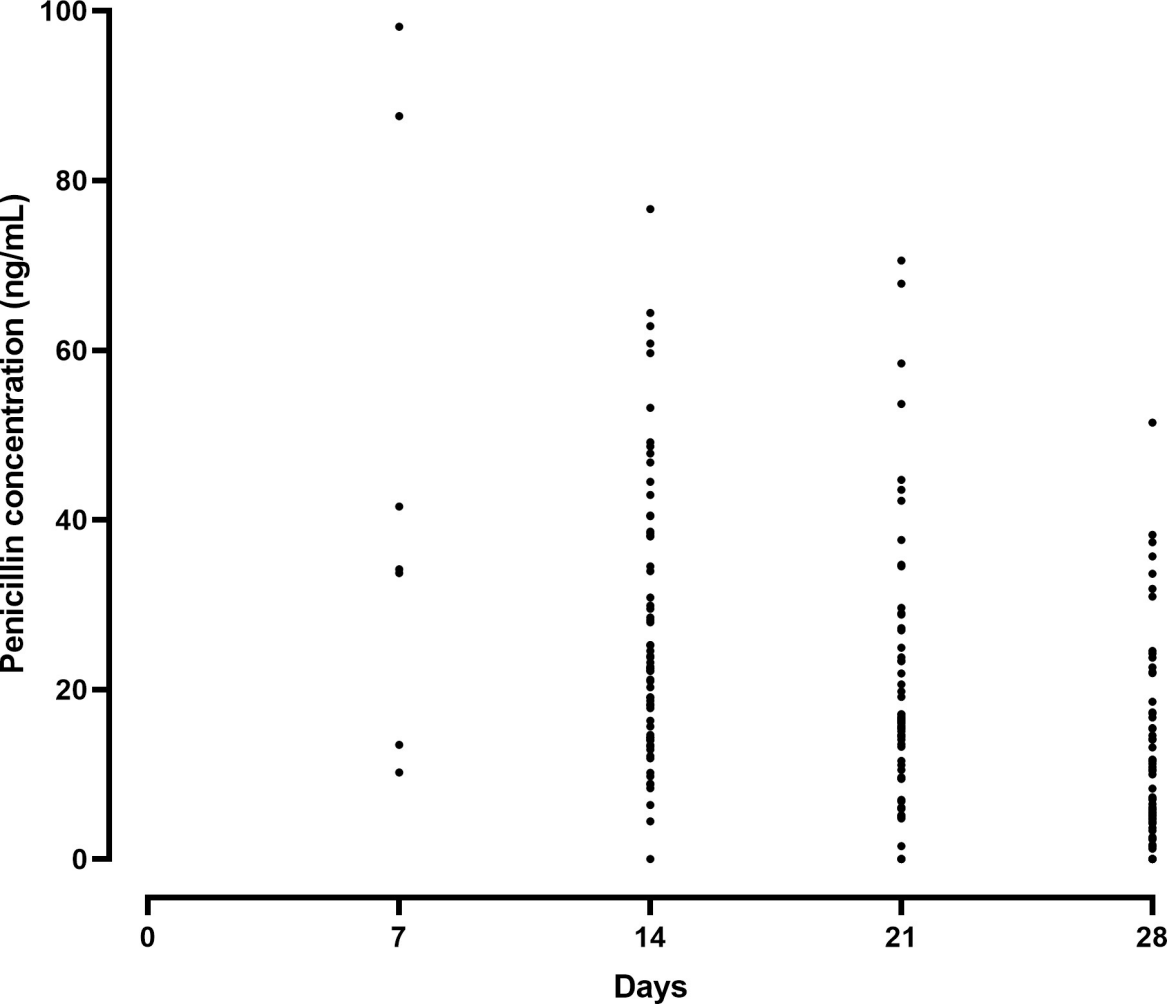
Measured penicillin concentrations following benzathine penicillin G injection in Ethiopian patients with rheumatic heart disease.

The remaining 74 patients had a total of 190 penicillin concentrations that were included in the PK model (see Fig 1 for raw data). Fifty (67.6%) were female and most (70.3%) resided in an urban area. The median and interquartile range (IQR) age, weight and BMI were 21 (17–30) years, 47 (37.5–53) kg and 18 (16–20) kg/m^2^, respectively. Only three participants had a BMI >25 kg/m^2^ and none had a BMI >30 kg/m^2^. One patient was receiving regular BPG doses every 21 days rather than every 28 days and nine were receiving the 0.6 MU dose. Of these, all weighed <27 kg, in accordance with the local dosing guidelines. The median (IQR) duration of RHD and regular secondary prophylaxis was 3 (1–4.7) and 2 (1–3.8) years, respectively. Seven (9.5%) reported at least one episode of sore throat during this single injection cycle, while 5 (6.8%) reported having had an ARF recurrence within the previous 12 months. One participant had co-existing HIV infection. Most (70%) of the participants showed a 100% adherence to monthly BPG injections. However, 30% of the participants had missed at least one dose during the 12 months prior to the study. Sixteen patients were diagnosed with RHD related cardiovascular complications (S1 Table).

The population pharmacokinetic model parameter estimates and bootstrap results for structural parameters for participants in the present study are shown in comparison with the same estimates derived from Indigenous Australian patients (Table 1). [16] The estimate for volume of distribution was lower (V/F = 54.8 [CI_95_ 53.8–66.3] versus 72.2 l.70kg^-1^) whilst the second, prolonged absorption half-life (t_1/2-abs2_) was longer (12.0 [CI_95_ 8.75–17.7] versus 8.88 days) in Ethiopian patients. The sensitivity analysis demonstrated little impact (<10%) on other fixed parameter estimates when t_½, abs-1_ was changed from 50 to 200% of the initial fixed value. As with previous models, the k_el_ of penicillin G was rapid (~100 fold faster than t_1/2-abs2_) and fixed to 1.32 h^-1^.70kg^-1^. Sensitivity analysis of this value resulted in no significant change in any parameter except V/F which was inversely proportional. This demonstrates that in this model k_el_ has scaling properties in the context of much slower absorption rate constants. The IIV for all parameters was higher in the Ethiopian model but the overall residual variability estimates for the model was similar (37% versus 35%; Table 1). Unlike the previous model, there was no significant effect of an elevated BMI on absorption when evaluated across units between 20 and 30 kg/m^2^.

**Table 1:** Parameterized final population pharmacokinetic estimates for structural model parameters for Ethiopian patients receiving benzathine penicillin G. Comparable parameter estimates for a similar structural model in Indigenous Australians are provided [16].

Parameter	Ethiopia	95% confidence intervals	Indigenous Australian urban population [16]
OFV	-6.357	-14.1773 (-53.5201–19.8077)	
k_el_ (h^-1^.70kg^-1^)	1.32 [FIX]	N/A	1.32 [FIX]
V/F (l.70kg^-1^)	54.8	53.8 (43.9–66.3)	72.2
t_½, abs-1_ (days)	0.455 [FIX]	N/A	0.455
t_½, abs-2_ (days)	12.0	11.7 (8.75–17.7)	8.88
Increase in t_½, abs-2_ with BMI ≥ 25 kg/m^2^ (%)	N/A	N/A	86.5
IIV in V/F	48	48 (35–60)	26
IIV in t_½, abs-1_	105	105 (65–174)	78
IIV in t_½, abs-2_	73	74 (47–133)	63
IOV in t_½, abs-2_	N/A (single dose)	N/A	30
r(t_½, abs-1_, t_½, abs-2_)	-1 [FIX]	N/A	-1 [FIX]
r(t_½, abs-2_, V/F_Z_)	-0.18	-0.105 (-0.65–0.466)	-0.746
RV (%)	37	36 (28–42)	35

kel (elimination rate constant), V/F (relative volume of distribution), t½, abs (absorption half-life), BMI (body mass index), IIV (inter-individual variability, IOV (inter-occasion variability) and RV (residual variability)

**Fig 2:**
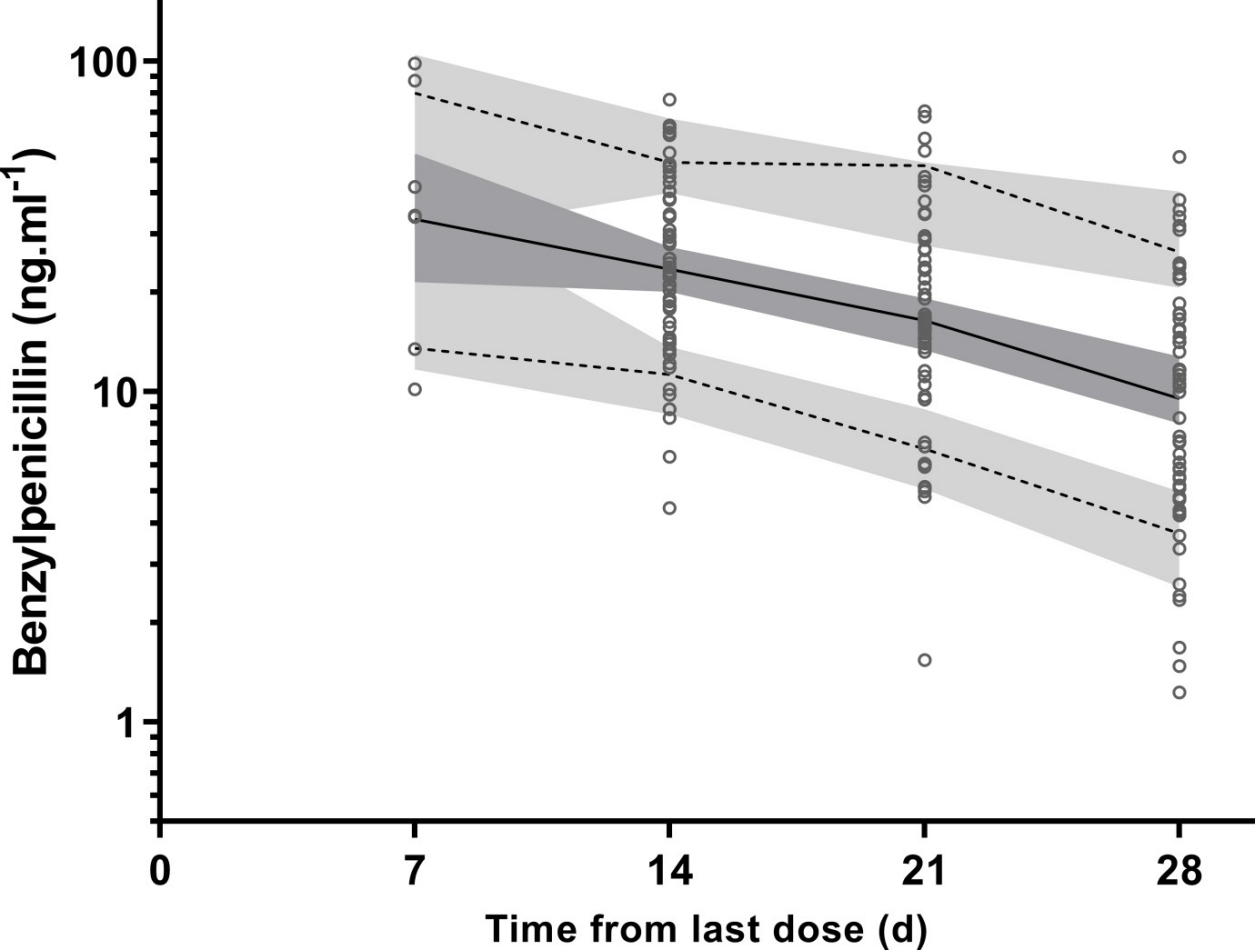
Visual predictive checks for plasma penicillin G concentrations (ng.mL^-1^ on log_10_ scale). Observed 50th (solid line), and 10th and 90th (dotted lines) percentiles within their simulated 95% confidence intervals (grey shaded areas) are shown with overlying the data points (○).

Plots of VPC with the actual 10^th^, 50^th^ and 90^th^ percentiles of observed data all within their respective CI_95_ are shown (Fig 2). Suitable predictive performance of the model was also demonstrated with goodness of fit plots (S1 Fig).

**Fig 3:**
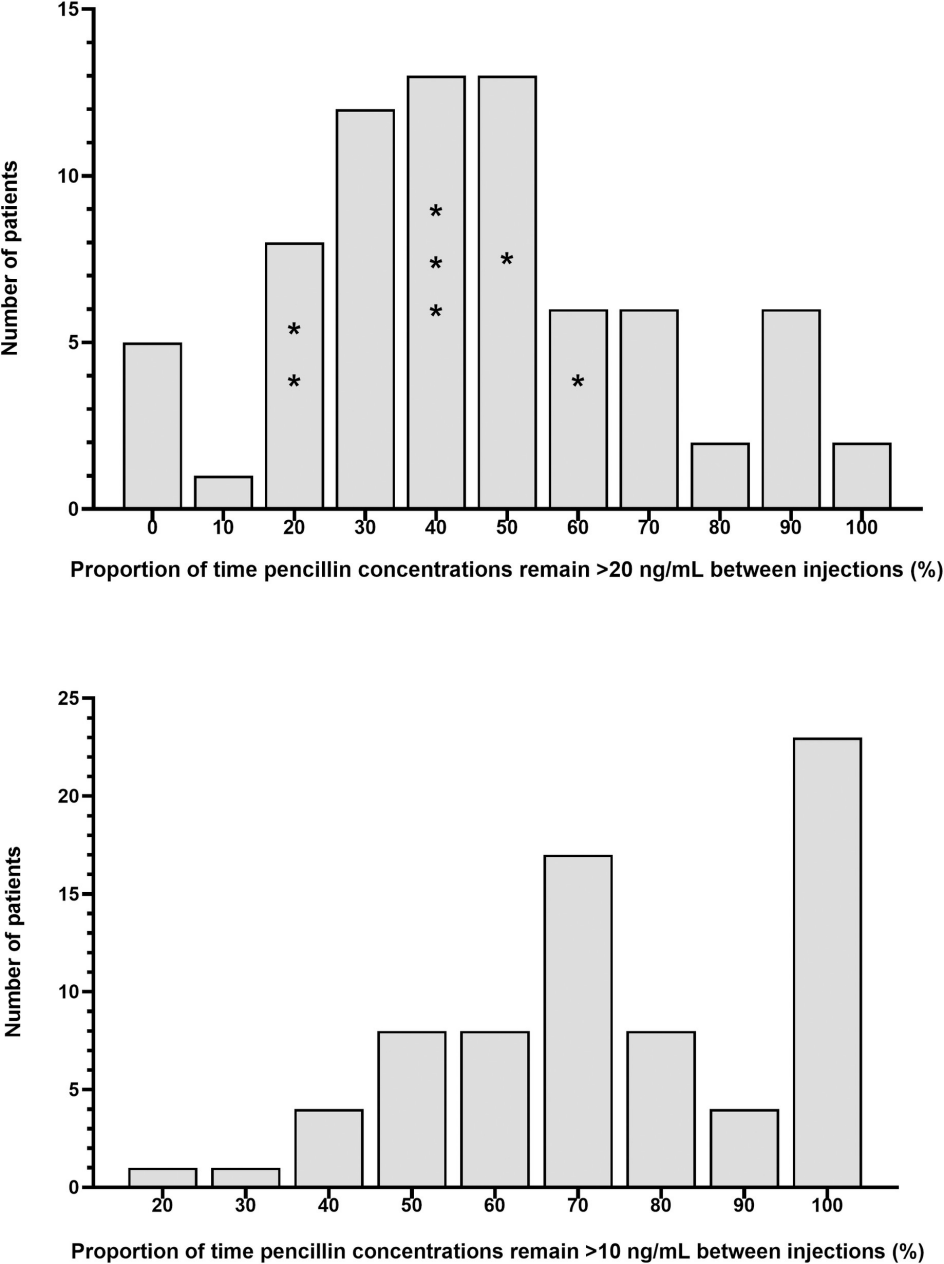
Histograms showing estimated time that penicillin concentrations remain greater than 20 ng/mL (Panel A) and 10 ng/mL (Panel B) following administration of intramuscular benzathine benzylpenicillin G to Ethiopian patients at risk of progressive rheumatic heart disease. Individuals with a self-reported episode of sore throat during the injection cycle are denoted (*).

Based on this model, a concentration-time profile was estimated for each individual, together with an estimate of time (in days) during each injection cycle where the plasma concentrations remained >20 ng/mL. The median (IQR) percentage of time for this PK outcome of interest was 42.5% (27.5–60; 12 days). As a further exploratory analysis, the median (IQR) proportion of time concentrations remained >10 ng/mL was 73% (58.5–99; 20 days). The spread of these estimates are shown in Fig 3A and 3B, respectively. Individuals self-reporting an episode of sore throat between injections are also shown in relation to time >20ng/mL. There was no significant difference between the mean values from participants that reported an episode of sore throat compared with those that did not (Student’s t-test, *P* = 0.65). The onset date and pharyngeal culture results for patients reporting sore throat were unavailable.

## Discussion

Our study revealed that most Ethiopian patients receiving BPG as secondary prophylaxis to prevent RHD do not maintain target concentrations for more than two weeks during each 4-weekly injection cycle. We have also shown that, compared with Indigenous Australian children and young adults, the V/F was lower and the t_1/2 abs-2_ was longer in Ethiopian patients. Although further studies are needed to confirm this, our data suggests that differences in BPG preparations and body composition differences between these two groups may contribute for the variations in BPG kinetics.

The result from this study accords with recent population PK studies of BPG in health military recruits [15] and Indigenous Australians receiving BPG as secondary prophylaxis for RHD. [16] These data add to the growing evidence base of contemporary studies highlighting the apparent pharmacological shortcomings of BPG and the urgent need to develop better long acting penicillin preparations for use in combatting RHD.

Although the structure of a prior population PK model from Indigenous Australians could be applied with significant bias to the present sparse dataset, there were substantial differences in the final parameter estimates in Ethiopian patients. The V/F was lower and the t_1/2-abs2_ was longer in Ethiopian patients compared with Indigenous Australian children and young adults. When compared with Indigenous Australians, the V/F was 31% lower (CI_95_ 9%-64%). A lower V/F corresponds to higher observed plasma concentrations for a given fat-free mass (FFM). One possible explanation for this observation is that there may be body composition differences between these two high risk populations. As with our previous model, we applied an accepted formula to estimate FFM which depends on gender, weight and height, but does not account for between population variability. Although between population variation in assessment of FFM has been reported, [28] the scale and directionality, and resulting effect on the model, of any possible differences between Ethiopians and Indigenous Australians remains speculative.

Another possible explanation for lower V/F (and higher concentrations for a given FFM) is that patients in the present study inadvertently received a dose greater than 900 mg. Variability in potency of BPG preparations exist, noting the absence of manufacturing standards. [29] A recent global quality study tested 35 different powdered BPG batches from 16 countries, including 17 batches from 7 sub-Saharan African countries (excluding Ethiopia), and demonstrated a median potency of 102% with a range of 95–108%. [18] Whilst we did not analyse the pharmaceutical quality of the generic preparation used in the present study, given the range of potency observed across the region, a relatively high dose in each vial compared to the nominal strength would contribute to the observed difference in V/F.

Differences in BPG preparations may also account for observed variability in the absorption kinetics from the site of injection. In the present study, we used a powdered formulation that was suspended in sterile water according to the manufacturer’s instructions, whereas in the study of Indigenous Australians, Bicillin L-A (Pfizer Australia Ltd, 1016.6mg/2.3mL) was administered. Although there are no direct PK comparisons of the more expensive, pre-suspended Bicillin L-A and the cheaper, powdered BPG preparations, important PK differences between powdered preparations have been observed in previous studies. [30] Variation in BPG crystal size and distribution, [31] has been observed in a recent BPG quality study, [18] which may have clinical implications and explain the differences in observed absorption profiles demonstrated here, as well as in previous publications. [30]

The present study had limitations. At the time the study was designed, the first sampling point was selected to be 14 days following injection. In future studies of BPG PK in Ethiopia and other high risk groups, the sampling schedule should be optimized to better reflect the complex absorption kinetics, with a richer sampling schedule during the first 14 days. However, we were able to estimate the most clinically relevant parameter, namely the slower absorption half-life, which determines the observed terminal half-life. Despite sparse data, we used a prior population PK model to generate a new model with adequate predictive performance to determine the key outcomes of interest with acceptable confidence. Reassuringly, sensitivity analyses of fixed parameters did not have a significant effect on model performance.

## Conclusion

The majority of Ethiopian patients receiving BPG as secondary prophylaxis to prevent RHD do not attain target concentrations for more than two weeks during each 4-weekly injection cycle, highlighting the limitations of current BPG strategies which may account for ARF relapses and RHD progression in this high risk population. The present study also highlights significant between-population PK variation and/or the possibility of important differences between BPG preparations which may have important implications for ARF/RHD control programs.

## Supporting information

S1 Fig Diagnostic plots of the population pharmacokinetic model. Observed versus population predicted plasma concentrations (A), observed versus individual predicted plasma concentrations (B), weighted residuals versus time (C) weighted residuals versus population predicted concentrations (D). The solid lines are lines of identity.
(TIF)

S1 Table Sociodemographic and clinical characteristics of study participants at Ayder Comprehensive specialized hospital, Mekelle, Ethiopia, between February and October, 2018. (DOCX)
