# Supplementary figures and images for: High risk of early sub-therapeutic penicillin concentrations after intramuscular benzathine penicillin G injections in Ethiopian children and adults with rheumatic heart disease
Source: PLoS Negl Trop Dis. 2021 Jun 11;15(6):e0009399. doi: 10.1371/journal.pntd.0009399 (PMC8195421; doi:10.1371/journal.pntd.0009399)

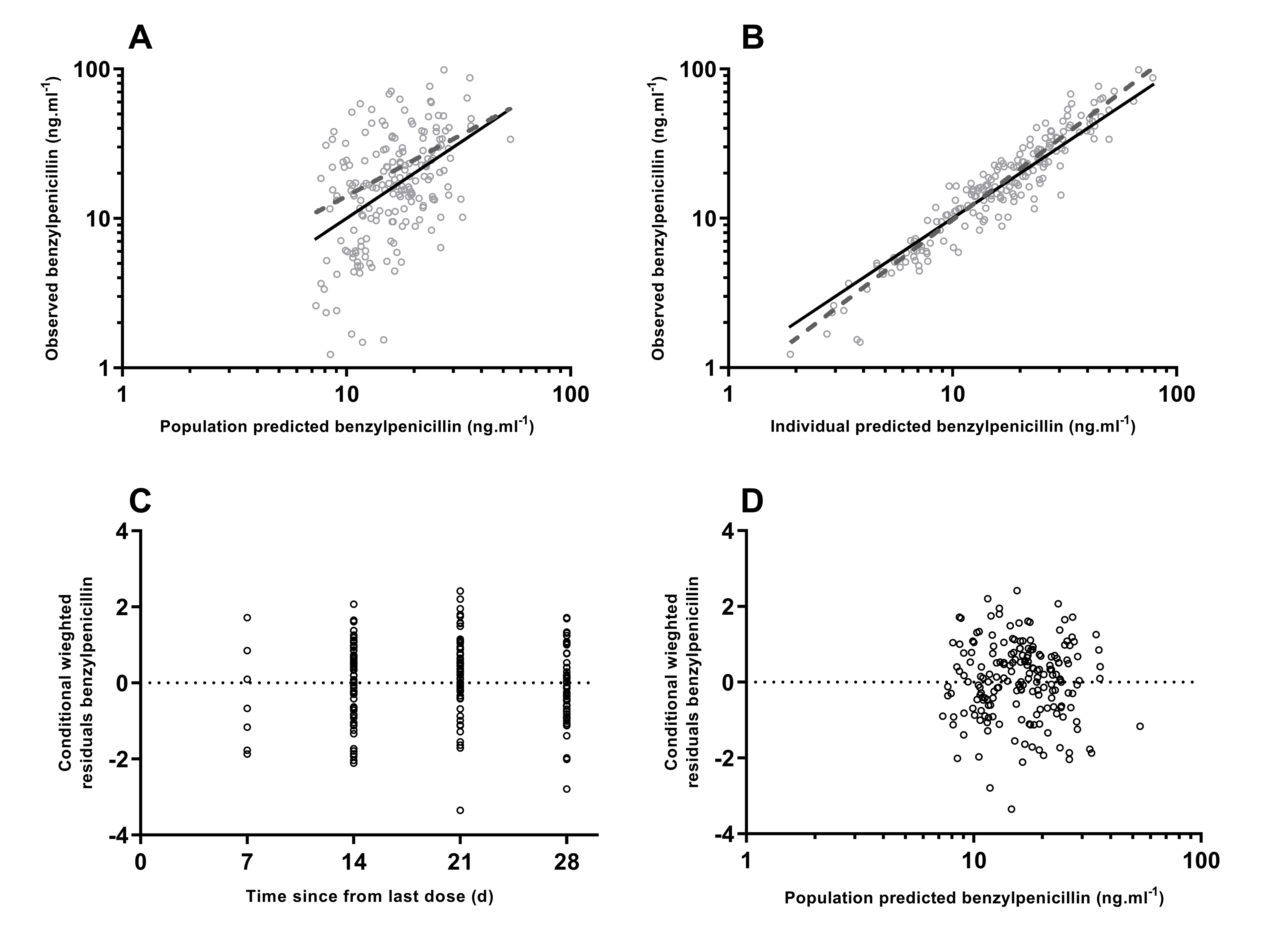

Supplement: S1 Fig — Observed versus population predicted plasma concentrations (A), observed versus individual predicted plasma concentrations (B), weighted residuals versus time (C) weighted residuals versus population predicted concentrations (D). The solid lines are lines of identity. (TIF) [file pntd.0009399.s001.tif]
