# Supplementary material for: High risk of early sub-therapeutic penicillin concentrations after intramuscular benzathine penicillin G injections in Ethiopian children and adults with rheumatic heart disease
Source: PLoS Negl Trop Dis. 2021 Jun 11;15(6):e0009399. doi: 10.1371/journal.pntd.0009399 (PMC8195421; doi:10.1371/journal.pntd.0009399)
Supplement: S1 Table — (DOCX) [file pntd.0009399.s002.docx]

| Characteristics | Frequency, n(%) | Characteristics | Frequency, n(%) |
| --- | --- | --- | --- |
| Age( years) ( mean + SE) | 23. 3 +1.4 | Weight (Kg) |  |
| 7-18 | 35(43.2) | <27 | 9 (11.7) |
| 19-30 | 29(35.8) | 27-40 | 20 (26.0) |
| >30 | 17(20.9) | 41-55 | 34(44.2) |
|  |  | >55 | 14(18.1) |
| Sex |  | BMI |  |
| Female | 53(66.3) | <18 | 31(50) |
|  |  | 18-27 | 31(50) |
| Residence |  |  |  |
| Rural | 55(68.8) |  |  |
| Urban | 25(31.2) |  |  |
| Education status |  | Home distance from health facility (km) |  |
| No formal education | 5(6.5) | <15 | 32(64.0) |
| primary school | 28(36.4) | 15-30 | 11(22.0) |
| high school | 30(39.0) | >25 | 7(14.0) |
| college | 14(18.2) | Adherence rate |  |
| Family size |  |  |  |
| <3 | 27(33.8) | <80% | 6(7.5) |
| 4-6 | 33(41.2) | 80-95 | 18(22.5) |
| >6 | 20(25.0) | 100% | 56(70.0) |
| Duration of RF/RHD (in years), mean +SE | 4.5+ 0.6 | Pharyngitis after BPG prophylaxis |  |
| <2 years | 31(38.8) | Yes | 8(10) |
| 3-5 years | 26(32.4) | Recurrence after BPG prophylaxis |  |
| 6-10 years | 19(23.8) | Yes | 6(7.5) |
| >10 years | 4(5) | Family history of ARF/RHD |  |
| Duration of BPG prophylaxis (in years), mean + SE | 3.5+ 0.5 | Yes | 2(2.4) |
| <1 | 33(42.8) | Skin infection |  |
| 2-5 | 28(36.4) | Yes | 4(4.9) |
| >5 years | 16(20.8) | Current RHD complication |  |
| BPG doses |  | Yes | 16(19.5) |
| 1.2 million units | 73(89) | Other comorbidities |  |
| 600,000 units | 9(11) | Yes | 3(3.7) |
| BPG injection missing in the past 12 months |  |  |  |
| Yes | 24(30) |  |  |
| Reason for Missing |  |  |  |
| Economic reasons | 8(10) |  |  |
| Awareness | 4(5) |  |  |
| Fear of injection | 4(5) |  |  |
| Inaccessibility of health facility | 5(6.3) |  |  |
| Others | 3(3.7) |  |  |
